# Diagnostic Efficacy of Sonography for Diagnosis of Ovarian Torsion

**Published:** 2014

**Authors:** Ayoob Rostamzadeh, Sam Mirfendereski, Mohammad Jafar Rezaie, Shohreh Rezaei

**Affiliations:** 1 Ayoob Rostamzadeh, Department of Anatomical Sciences, Cellular & Molecular Research Center, Faculty of Medicine, Kurdistan University of Medical Sciences, Sanandaj, Iran.; 2 Sam Mirfendereski, Department of Radiology, Imam Reza Hospital Research Center, Faculty of Medicine, Kermanshah University of Medical Sciences, Kermanshah, Iran.; 3 Mohammad Jafar Rezaie, Cellular and Molecular Research Center, Department of Embryology, Faculty of Medicine, Kurdistan University of Medical Sciences, Sanandaj, Iran.; 4 Shohreh Rezaei, Deputy of Medical Research, Kurdistan University of Medical Sciences, Sanandaj, Iran.

**Keywords:** Torsion, Sensitivity, Specificity, Ultrasonography

## Abstract

***Objectives:*** Misdiagnosing ovarian torsion is now suggested as an important issue in clinical setting. The aim of this study was to determine the diagnostic accuracy of sonography for ovarian torsion.

***Methods*** ***:*** In this study 323 women with acute pelvic pain with highly suspected ovarian torsion signs and symptoms attending Imam Reza Medical Center in Kermanshah between 2011 through 2012 were included and underwent a transabdominal sonography (2-5 MHz probes). Then findings of sonography were compared with laparatomy.

***Results*** ***:*** The ultrasound correctly diagnosed 72.1% of ovarian torsion and missed 27.9% of them (false negatives). However, one free subject (0.4%) was misclassified as ovarian torsion (false positive). There was a strong correlation between sonography and laparatomy with a kappa value of 84.0%. The sensitivity and specificity of sonography were 72.1% and 99.6%, respectively. Sonography had a positive predictive value of 96.9%, a negative predictive value of 95.9%, and a total accuracy of 96.0% for detection of ovarian torsion.

***Conclusion:*** Sonography appears to be an excellent method to evaluate patients with suspected ovarian torsion. Abnormal blood flow detected by sonography is highly predictive of ovarian torsion and is therefore useful in the diagnosis of this phenomenon.

## INTRODUCTION

Misdiagnosing ovarian torsion is now suggested as an important issue in clinical setting that is suggested to be related to the variety of its clinical manifestations.^1^ Clinically, this phenomenon can be manifested by severe pain in lower abdomen, occasionally with nausea and vomiting, however pain referring to the position of other organs such as kidneys and pelvic-related systems so clinical presentation of ovarian torsion is variable and often misleading. Also, physical examination is non-specific and peritoneal irritability may be present or absent.^1^^-^^3^

In this regard, differential diagnosis of this disorder is wide and consists in multiple other causes of abdominal pain including other gynecological, gastrointestinal, and urinary tract diseases.^4^ Besides, if complete ovarian torsion is undiagnosed and untreated, it can lead to infertility, ovarian necrosis with peritonitis, loss of ovary, and death and thus delaying in its diagnosis may lead to life-threatening consequences.^5^ Because of selecting the affected patients for emergent surgery, its timely and minute diagnosis is vital that remained challenging among specialists. Meanwhile, despite recent development of imaging techniques, medical imaging modalities remain controversial. Utility of conventional ultrasonography for diagnosis of ovarian torsion has been now questioned. This imaging procedure can successfully detect adnexal lesions and ovarian enlargement.^6^

However, in suspected patients to ovarian torsion, the presence of normal-appearing ovaries does not rule out its diagnosis. For improvement of ultrasonography application for detection of ovarian torsion, Doppler flow study has been employed aimed to assist clinicians in reaching the more accurate diagnosis of ovarian torsion.^7^ Because the accuracy of conventional sonography remains controversial in diagnosis of ovarian torsion, the aim of the present study was to examine the diagnostic performance of sonography for detection of ovarian torsion compared with surgical observation.

## METHODS

This study was conducted in the Imam Reza Medical Center in Kermanshah city. All female patients younger than 40 years of age who presented with acute pelvic pain with highly suspected ovarian torsion in 2011 through 2012 and for whom laparatomy were performed within less than 6 hours of the ultrasound assessment were included. The informed consent was obtained from patients. The main inclusion criteria included raising the possibility of ovarian torsion in terms of clinical signs and also agreement with the participation in the study. The study was approved by Research Council of Kermanshah University of Medical Sciences** (**Code of project: 91236).

Failure to perform surgery on the patients was the study exclusion criterion. Sample size was determined at 95% confidence interval, 5% precision and 70.0% expected prevalence of diagnosing ovarian torsion in sonography assessment from previous study and found to be at least 323 patients. Patients underwent a transabdominal sonography (Aulsion GE) using 2-5 MHZ probes by an ultrasound specialist. Benchmark of positive test was ovarian enlargement with no arterial or venous blood flow. The findings of sonography were compared with laparatomy findings. The data were collected by chart review for all patients. All baseline variables including demographics and clinical manifestations were recorded. Sensitivity, specificity, and predictive values were determined by cross-tabulation of the results of ultrasound and the surgical findings of ovarian torsion as the gold standard. Based on this tabulation, the sensitivity, specificity and positive predictive values were computed for ultrasound. In addition, the level of agreement between the ultrasound diagnosis and the surgical diagnosis was determined by calculating the kappa coefficient. The data were analyzed using SPSS version 19.0. All the statistical tests were considered significant at P≤0.05.

## RESULTS

The average age of patients was 26.3±7.8 years. Of cases with abdominal pain, 43 (13.3%) were confirmed as cases of ovarian torsion by surgery and other surgical diagnoses were appendicitis (24.8%), hemorrhagic cyst (22.9%), ectopic pregnancy (21.1%), and others (18.0%). The highest and the lowest mean age was specified to women suffering from ovarian torsion (24.9 ± 8.0 years) and hemorrhagic cyst (26.9 ± 7.8 years). Twenty eight women (8.7%) were pregnant who suffered the most from ovarian torsion compared with non-pregnant women (35.7% versus 11.1%, p < 0.001).

The ultrasound correctly diagnosed 72.1% of ovarian torsion cases and missed 27.9% of these cases (false negatives). However, one free subject (0.4%) was misclassified as ovarian torsion cases (false positive), there was a strong correlation between sonography and surgery with a kappa value of 84.0%. The sensitivity and specificity of sonography for diagnosing ovarian torsion were determined 72.1% and 99.6%, respectively. Also, sonography had a positive predictive value (PPV) of 96.9%, a negative predictive value (NPV) of 95.9%, and a total accuracy of 96.0% for detection of ovarian torsion.

Of all diagnosed ovarian torsion surgically, 26 (60.5%) was detected in the right-sided and 17 (39.5%) in the left-sided that sonography assessment led to diagnose 18 in the right-sided and 13 in the left-sided, respectively (p = 0.61). Sixteen (37.2%) detected torsions had primary pattern and 62.8% had secondary pattern that sonography assessment was successful in detecting 81.2% of primary torsions and 66.7% of secondary torsions, respectively (p = 0.48). Ovarian echogenicity was increased in 7.4% and remained normal in 93.6%. Echogenicity was significantly higher in those with ovarian torsion than non-affected ones (p < 0.001). Based on sonography findings, blood flow was not revealed in 9.9% of affected ovaries. Of 43 diagnosed torsions surgically, 72.1% had no evidence of arterial or venous blood flow, while only one of the subjects without ovarian torsion was diagnosed not to have blood flow (p < 0.001). The mean diameter of affected ovaries by torsion was significantly higher than that measured in normal ovaries (72.4 ± 48.7 mm versus 16.00 ± 9.8 mm, p < 0.001). Finally, among 43 diagnosed partial torsion, 32 (74.4%) were completed and 11 (25.6%) were partial that 87.5% and 27.3% had no evidenced blood flow in sonography views respectively. There was a significant correlation between ovarian torsion completeness and ovarian blood flow (p < 0.001).

## DISCUSSION

This study sought to determine the value of sonographic assessment for diagnosis of ovarian torsion compared to surgical observation. According to our findings, sonographic diagnosis of ovarian torsion had overall accuracy of 96.0% with the sensitivity of 72.1% and the specificity of 99.6%, respectively. Comparing our results with the previous reported findings confirm higher obtained diagnostic value of sonography compared with some studies and lower value compared with others in our survey. In a similar study by Mashiach and colleagues, sonography had diagnostic accuracy of 74.6% for ovarian torsion.^8^ However, in a study by Graif et al. a 100% sonographic sensitivity and 93% specificity for space-occupying disease of the ovary were obtained with a positive predictive value of 88% for the diagnosis of ovarian torsion in both in childhood and adolescence.^9^ Because of the variety of reported diagnostic performance for sonography in diagnosing ovarian torsion, it can be suggested that an ultrasound exam may be used to make a diagnosis in conjunction with clinical parameters; however, this will be most difficult in patients with ovarian torsion because of its non-specific symptoms.^10^ The only specific sonographic sign of torsion is demonstration of multiple follicles (8-1 2 mm in size) in the cortical portion of a unilaterally enlarged ovary. This multifollicular enlargement was attributed to transudation of fluid into the follicles as part of the congestion of the ovary from circulatory impairment. This feature has been detected by careful examination in up to 74% of torsion cases.^11^ It has been also shown that sonographic features consistent with diffuse swelling of the ovarian parenchyma and follicular enlargement in the cortical zone can be detected in most patients and can be considered highly suggestive for torsion of the ovary.^9^ Thus in combination of imaging assessments and clinical findings, this more specific sign should be considered to obtain more accurate diagnosis. In total, in the setting of a specialized ultrasound unit, sonographic diagnosis of ovarian torsion had high accuracy compared with previous reports.

In our survey, about 72% of affected women with ovarian torsion were diagnosed to have abnormal blood flow, while this rate in non-affected ones was estimated only 0.4%. On the other hand, abnormal flow detected by sonography seems to be highly predictive of ovarian torsion and therefore can be valuable in the diagnosis of ovarian torsion that similarly pointed by Pena et al.^12^ However, the sensitivity was only 72.1% which indicates that the normal sonographic findings do not rule out ovarian torsion.

The presence of central venous flow and flow in the vascular pedicle indicates ovarian viability.^13^ In addition, Mashiach et al. showed that abnormal ovarian blood flow was the most diagnostically accurate isolated sonographic sign with PPV 80.0%.^8^ The specificity of this procedure was found to be 100% in some other studies.^14^ Thus, considering abnormal ovarian blood flow as a high sensitive criterion is useful for accurate detection of ovarian torsion that should be applied beside other diagnostic imaging parameters for this phenomenon.

## CONCLUSION

According to the study findings, in case of clinical doubtfulness to torsion, ovarian sonography is strongly recommended. Also, if volume of ovary was increased simultaneously, examination by using color Doppler is also necessary. In total, According to high specificity of sonography, in cases with increased volume of ovary and lack of flow, ovarian torsion is strongly considered as diagnosis and the patient should be candidate for surgical management. However, because of rather low sensitivity of this diagnostic technique, in cases with increased volume of ovary but with normal flow, partial torsion may be considered and the follow-up of patient for determining necessity of emergency surgery should be programmed.
